# Health Implications of Lipedema: Analysis of Patient Questionnaires and Population-Based Matched Controls

**DOI:** 10.3390/life14030295

**Published:** 2024-02-22

**Authors:** Sally Kempa, Mascha Gross, Dmytro Oliinyk, Andreas Siegmund, Martina Müller, Lukas Prantl, Hauke C. Tews

**Affiliations:** 1 Department of Plastic, Hand, and Reconstructive Surgery, University Hospital Regensburg, 93053 Regensburg, Germany; 2 Department of Internal Medicine I, Gastroenterology, Hepatology, Endocrinology, Rheumatology, and Infectious Diseases, University Hospital Regensburg, 93053 Regensburg, Germany

**Keywords:** lipedema, obesity, questionnaire, pain, depression

## Abstract

We conducted a comparative study involving 39 female patients with lipedema and group-matched controls at a ratio of 1:5. The primary survey tool was the German Health Update (GEDA 2019/2020-EHIS) questionnaire, which was developed by the Robert Koch Institute (RKI), Germany. The secondary survey tool was the German Pain Questionnaire. The prevalence of hypertension (*p* = 0.041) and high blood lipids (*p* = 0.024) was lower in the lipedema group compared to the control group. General health and well-being indicators demonstrated lower overall health ratings (*p* < 0.001) and higher physiotherapy use in patients with lipedema (*p* = 0.016). Mental health assessment revealed higher depression prevalence and severity (*p* = 0.001), together with a lower number of close contacts (*p* = 0.032). Furthermore, patients with lipedema experienced higher levels of pain (*p* < 0.001) and more significant pain-related disability in daily activities (*p* < 0.001) than controls. Correlation analysis among patients with lipedema showed a positive correlation between pain severity and depressive symptoms (ρ = 0.612, *p* < 0.001) and a moderate positive correlation with impaired health-related quality of life (ρ = 0.418, *p* = 0.010). In summary, our findings highlight significant differences in health and well-being between patients with lipedema and matched controls, especially in overall, metabolic, and mental health, as well as pain perception. The findings emphasize the need for a validated lipedema-specific questionnaire and a multidisciplinary treatment approach with a combination of physical therapies, lifestyle adjustments, and psychological strategies.

## 1. Introduction

Lipedema is a chronic condition that mainly affects women and is characterized by disproportionate, painful, and persistent enlargement of adipose tissue, particularly in the lower extremities. The condition deeply impacts daily life and well-being [1,2,3], stemming from not only distorted body image but also physical pain [4,5,6,7,8,9] and psychological distress (such as depression or eating disorders) [10], which contributes to a high individual and public health burden.

The distinction between lipedema and obesity is crucial because there are currently no specific objective signs to differentiate between the two diseases. Unlike obesity, which responds to changes in diet, abnormal lipedema fat is resistant to weight loss, often leading to a disproportion between a slim upper body and an enlarged lower body. Patients with lipedema often have a higher body mass index (BMI) but do not exhibit the typical cardiovascular and lipid profile alterations associated with obesity [11]. Although these differences are often cited in the literature, there are few controlled studies that have been able to objectify these differences. To further challenge the differential diagnosis, lipedema is often complicated by obesity later in life, so it is necessary to look at the history of symptoms to make an accurate diagnosis [12].

To evaluate patients with lipedema, various questionnaires such as WHOQOL-Bref [4,7,13,14,15], RAND-36 [16,17], EuroQol-5D-3L [17], the 36-Item Short Form Health Survey (SF-36) [8,12,18], and the Freiburg Quality of Life Assessment for lymphatic disorders (FLQA-lk) [18] have been employed. However, no health-related quality-of-life instrument has been validated specifically for lipedema [19]. In addition, the disorder is dynamic, yet there are no validated tools to monitor longitudinal changes in lipedema.

Because of this gap, our work aims to improve the scientific data on lipedema by comparing the health-related quality of life in patients with lipedema to those with obesity, hypothesizing significant differences in their clinical presentations.

## 2. Patients and Methods

### 2.1. Study Design and Ethical Approval

This questionnaire-based, cross-sectional survey was approved by the local Ethics Committee, and all participants provided written informed consent. Formal and documented ethical approval was obtained (reference number 22-3163-101, University of Regensburg).

### 2.2. Selection of Patients with Lipedema and Controls

In this study, we conducted a comparative study involving 39 patients with lipedema. Participants were identified among individuals who either had a previous diagnosis of lipedema or were suspected of having the condition. The diagnosis was confirmed by a team of experienced physicians at the University Hospital Regensburg, Germany, through comprehensive physical examination and medical history. This process ensured that all women in the lipedema group met the inclusion criteria outlined in Table 1 of our study. The exclusion criteria included history of previous liposuction treatments, asymmetrical legs, and pregnancy. Participation in this study was voluntary, and no incentives were offered.

To establish the control group, we compared these patients with participants from the German Health Update study, a large-scale health telephone survey conducted on behalf of the Federal Ministry of Health in Germany [20]. It included a total of 23,001 individuals aged 15 and over living in private households in Germany. This extensive dataset was made available for this study as a scientific use file by the Robert Koch Institute in Germany. Our control group was selected using SPSS software, employing the SPSS case–control matching feature, matching at a ratio of 1:5, based on the participant’s age at the time of participation, classed as group 1 (18–29 years), group 2 (30–44 years), group 3 (45–64 years), and group 4 (65 years or older), and on BMI groups using the WHO body mass index classification [21].

### 2.3. Questionnaires

#### 2.3.1. German Health Update (GEDA 2019/2020-EHIS) Questionnaire

The primary survey tool was the German Health Update (GEDA 2019/2020-EHIS) questionnaire [22], which was developed by the Robert Koch Institute (RKI) in Germany. This questionnaire is widely recognized for its comprehensiveness in assessing general health status and has been used extensively in public health research in Germany. Each module aimed to provide a comprehensive understanding of the participants’ health status, healthcare usage, and lifestyle factors that impact their health. Further details of the methodology of GEDA 2019/2020-EHIS are extensively described in previous publications [22]. A relevant selection of the following modules was used in the lipedema group.

General health status: The 12-month prevalence of chronic diseases was assessed by asking participants to report any long-term illnesses or chronic health issues they experienced in the past year, explicitly excluding temporary health problems by answering “yes”, “no”, or “I don’t know” to a list of specific diseases and conditions. Subjective general health status was assessed by selecting one of five given answer options: “excellent”, “good”, “fair”, “poor”, or “very poor”.

Prevention and health-related behavior: This module included questions regarding the utilization of various healthcare services including physiotherapy during the last 12 months with the following answer options: “yes”, “no”, or “I don’t know”.

Mental health and social support: For recording mental health, this survey used the internationally established 8-item Patient Health Questionnaire (PHQ-8) [23]. The presence of depressive symptoms was assumed from a scale sum value of at least 10 of the maximum 24 points. The number of close contacts was determined by the question “How many people are so close to you that you can rely on them when you have serious personal problems?” by answering “None”, “1 or 2”, “3 to 5”, “6 or more”, or “I don’t know”.

Functional aspects of health: Pain and pain-related impairment in daily activities were evaluated by the following questions: “Concerning physical pain: How severe has your pain been in the past 4 weeks?” and “To what extent has the pain prevented you from carrying out everyday activities at home and at work in the past 4 weeks?”

#### 2.3.2. German Pain Questionnaire (DSF, Deutscher Schmerzfragebogen)

Furthermore, to gain a deeper understanding of the pain experienced by patients with lipedema in their lower extremities, we included the modules on localization, description, and treatment of the pain from the validated German Pain Questionnaire (DSF, Deutscher Schmerzfragebogen) [24]. 

The participants with lipedema answered the questionnaires using the online SoSci Survey software tool version 3.5.01 (Munich, Germany) with a return rate of 76.4%, whereas controls were interviewed by telephone as part of the GEDA 19 study with a response rate of 21.6% [20].

### 2.4. Data Collection and Analysis

Questionnaires were manually added to the database. The data were analyzed using SPSS version 26.0 for Mac (SPSS Inc., Chicago, IL, USA). Descriptive statistics and frequencies were calculated. The frequencies of categorical variables were compared using Pearson χ^2^ or Fisher’s exact test, when appropriate. Correlations between the questionnaire variables were assessed using Spearman correlations. A value of *p* < 0.05 was considered significant.

## 3. Results

### 3.1. Clinical Characteristics of the Study Cohort

In total, 39 female lipedema patients, averaging 46.8 years old, predominantly between 45 and 64 years, responded to the survey. They were group-matched 1:5 with 195 controls based on age, sex, and BMI. Clinical stages among lipedema patients included 9 in stage I, 21 in stage II, and 9 in stage III. A family history of lipedema was reported in 64% of patients, with 54% noting onset during puberty. The clinical and demographic features are detailed in Table 2.

### 3.2. Metabolic Health Comparisons

**Figure 1:**
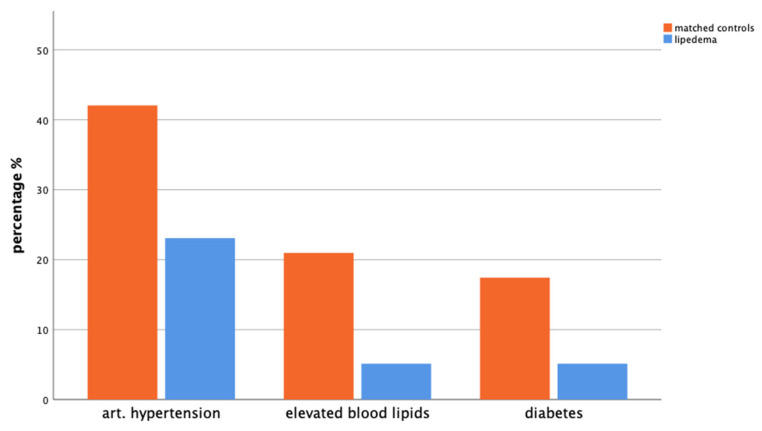
Bar chart presenting GEDA-19 questionnaire results for the presence of arterial hypertension (*p* = 0.041), elevated blood lipids (*p* = 0.024), and diabetes (*p* = 0.089) in the past 12 months in matched controls (orange, left) and patients with lipedema (blue, right).

When comparing the prevalence of metabolic diseases in the past 12 months, 42.1% of the control group reported hypertension, 21.0% had elevated blood lipids, and 17.4% had diabetes. In the lipedema group, the prevalence was 23.1% for hypertension (*p* = 0.041), 5.1% for elevated blood lipids (*p* = 0.024), and 5.1% for diabetes (*p* = 0.089) (Figure 1).

### 3.3. General Health and Well-Being Indicators

**Figure 2:**
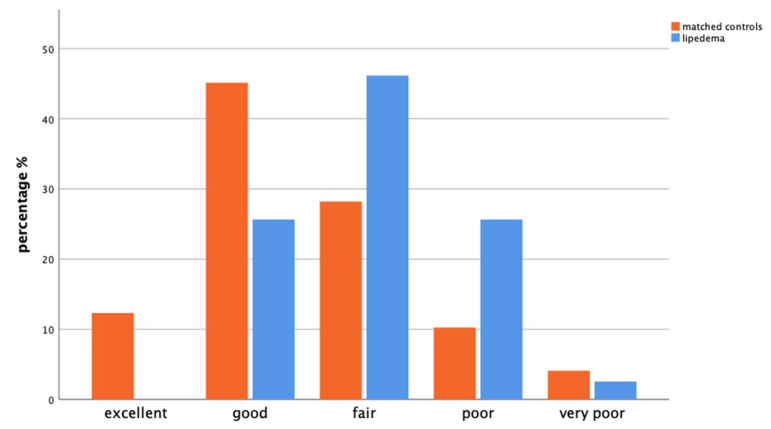
Bar chart showing the general health status of matched controls (orange, left) versus patients with lipedema (blue, right) from the GEDA-19 questionnaire (*p* < 0.001).

Comparison of the self-assessment of general health status showed significant differences between the two groups (*p* < 0.001). From the matched controls, 12.3% reported their health as excellent, 45.1% as good, 28.2% as fair, 10.3% as poor, and 4.1% as very poor. In contrast, no patients with lipedema rated their health as excellent, 25.6% rated it as good, most 46.2% rated it as fair, 25.6% rated it as poor, and 2.6% rated their health as very poor (Figure 2). 

Although differences were observed in the frequency of work absences due to illness, they were statistically not significant (*p* = 0.146). In the control group, 25.2% reported absences of 1–7 days, 20.6% for 8–14 days, 27.1% for 15–30 days, and 24.3% for 31–180 days. Only a small fraction (2.8%) reported absences longer than 180 days. Within the lipedema group, 11.5% reported absences lasting 1–7 days, but a significant proportion experienced longer absences: 26.9% for 8–14 days, 34.6% for 15–30 days, and 15.4% for 31–180 days. Notably, the percentage of individuals in the lipedema group reporting absences longer than 180 days (11.5%) was higher compared to the control group. 

Regarding use of physiotherapy services over the past 12 months, 87.2% of patients with lipedema reported visiting a physiotherapist during this period. In contrast, only 37.4% of the control group reported using physiotherapy services (*p* = 0.016).

### 3.4. Mental Health Assessment and Social Support

**Figure 3:**
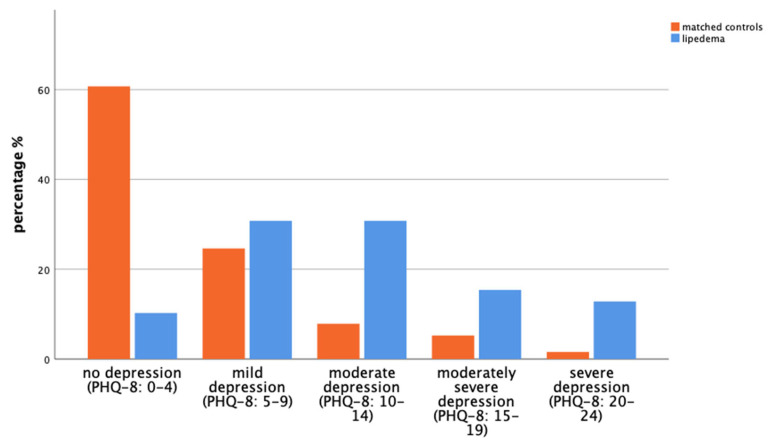
The bar chart illustrates the disparity in depressive symptoms between matched controls (orange, left) and patients with lipedema (blue, right) based on the PHQ-8 scores of the GEDA-19 questionnaire (*p* = 0.001).

In total, 43.6% within the lipedema group and 18.5% within the control group reported experiencing depression within the last 12 months (*p* = 0.001), which is corroborated by the Patient Health Questionnaire PHQ-8 questionnaire results. We observed a higher prevalence and severity of depressive symptoms in the lipedema group (Figure 3), where only a small fraction (10.3%) reported no depressive symptoms compared with most matched controls (60.7%) who reported no depressive symptoms (PHQ-8 score 0–4). For mild depressive symptoms (PHQ-8 score 5–9), 24.6% of the control group and 30.8% of the lipedema group were affected. Moreover, 30.8% of the lipedema group reported moderate depressive symptoms (PHQ-8 score 10–14), compared with 7.9% in the control group. Furthermore, the prevalence of more severe depressive symptoms was notably higher in the lipedema group. While 5.2% of controls reported moderately severe symptoms (PHQ-8 score 15–19), this was the case for 15.4% of those with lipedema. Additionally, severe depressive symptoms (PHQ-8 score 20–24) were reported by 1.6% of the control group, compared with 12.8% of the lipedema group.

**Figure 4:**
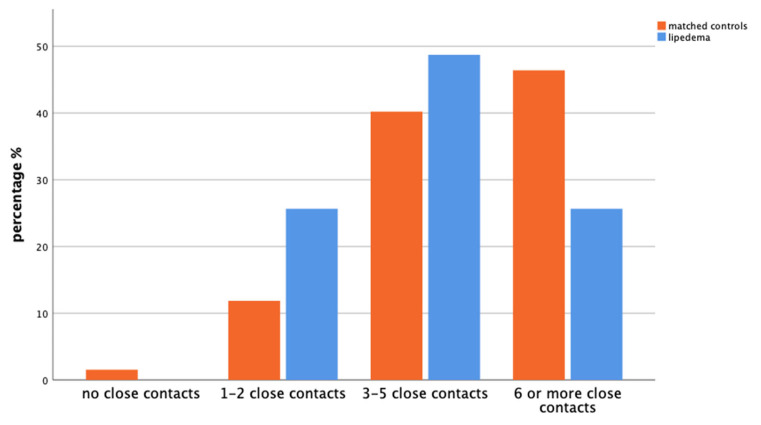
Bar chart with comparison of the percentage of matched controls (orange, left) and patients with lipedema (blue, right) based on the answers regarding the number of close contacts of the GEDA-19 questionnaire (*p* = 0.032).

Regarding the number of close contacts (*p* = 0.032), in the control group, 11.9% reported having only 1–2 close contacts, whereas the majority indicated a higher number of close contacts, with 40.2% having 3–5, and 46.4% having 6 or more. Only 1.5% of the control group reported having no close contacts. In contrast, the distribution among patients with lipedema was more evenly spread across the categories: 25.6% reported having 1–2 close contacts, 25.6% reported having 6 or more. The largest proportion, 48.7%, reported having 3–5 close contacts. Notably, none of the patients with lipedema reported having no close contacts (Figure 4).

### 3.5. Impact of Lipedema on Pain Levels and Daily Functioning

**Figure 5:**
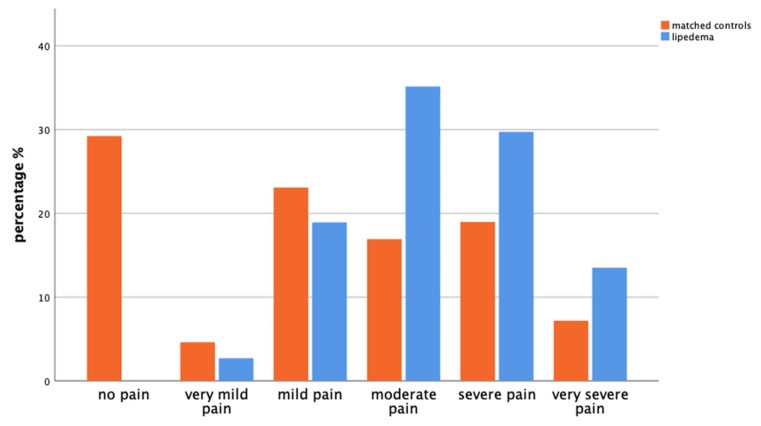
Bar chart showing the distribution of pain intensity experienced by matched controls (orange, left) and patients with lipedema (blue, right) in the last 4 weeks according to their answers of the GEDA-19 questionnaire (*p* < 0.001).

Patients with lipedema experience higher levels of pain (*p* < 0.001) and more significant disability in daily activities (*p* < 0.001) compared with controls. In the control group, 29.2% reported no pain, whereas 7.2% experienced very severe pain. On the other hand, none of the individuals with lipedema reported being free from pain; 13.5% reported very severe pain (Figure 5).

**Figure 6:**
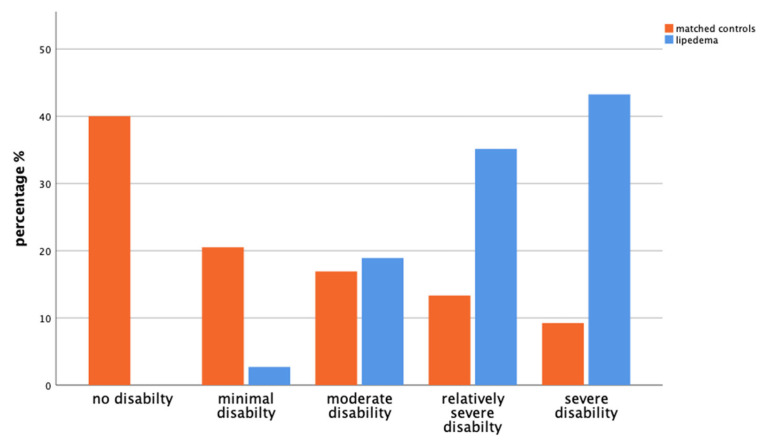
The bar chart displays the extent to which matched controls (orange, left) and patients with lipedema (blue, right) experience disability in daily activities due to pain over the last four weeks according to the GEDA-19 questionnaire (*p* < 0.001).

In terms of pain-related impairment in daily activities, 40% of the control group reported no disability from pain, while a gradual decrease in percentages was seen with increasing severity, with 9.2% reporting severe disability. In the lipedema group, the percentages significantly increased with severity, with the majority (43.2%) reporting severe disability, indicating a higher impact of pain on daily activities in the lipedema population (*p* < 0.001) (Figure 6).

Table 3 provides an overview of lower extremity pain characteristics reported by 39 patients with lipedema. This indicates that most patients experienced pain both at rest (79.5%) and with movement (87.1%). Various pain sensations were reported, with the most frequent being oppressive (64.1%), followed by pulling (56.4%), dull (51.3%), stabbing, awful, or miserable (each 43.6%). The intensity of pain at the moment of the survey on a numeric rating scale of 0–10 showed mean pain values over the last 4 weeks of 4, a maximum of 8, and a tolerable pain level with adequate treatment reported of 4. Correlation analysis among lipedema patients showed a positive correlation between pain severity and depressive symptoms (ρ = 0.612, *p* < 0.001) and a moderate positive correlation with impaired health-related quality of life (ρ = 0.418, *p* = 0.010).

Patients with lipedema reported various methods to improve circulation, reduce swelling, and thus relieve their pain. Many patients found relief from activities such as walking, swimming, light exercise, water aerobics, and yoga. Techniques such as lymphatic drainage, and the use of a lymphatic drainage devices were frequently mentioned. The use of compression garments or stockings was beneficial. Elevating the legs and taking rest breaks were frequently mentioned. Some patients found relief by applying cold water to their legs. Adequate hydration and a special diet, e.g., a ketogenic diet, were also mentioned as helpful. Distraction techniques, including talking, meditation, and sexual activity, were mentioned as methods of coping with pain. Some patients reported pain relief from taking diuretics such as torasemide and supplements. On the other hand, patients with lipedema also reported several factors that exacerbated their pain. Cold, heat, humid weather, and weather changes were frequently mentioned. Both extreme heat and cold aggravated the pain. Prolonged sitting, standing, walking, climbing stairs, bending, kneeling, and physical exertion in general were cited as aggravating factors. Activities such as house cleaning or other physical labor also exacerbated pain. A poor diet, particularly a high proportion of sugar and white flour, and alcohol consumption aggravated the pain. Emotional stress was repeatedly mentioned as increasing pain. Swelling of the extremities, physical pressure on the legs, and even touching intensified the pain. Long periods of working at a computer screen and incorrect lying positions were also mentioned. Interestingly, some patients reported that manual lymphatic drainage, if not performed correctly, exacerbated pain. The lack of compression on the legs was cited as a factor that can exacerbate pain, especially in hot and humid weather. These reports emphasize a combination of complex physical decongestive therapy, lifestyle adjustments, and psychological coping strategies to manage the pain associated with lipedema.

## 4. Discussion

In our comparative study, we examined lipedema-specific domains of a subset of questionnaires related to health-related quality of life (QoL) in both lipedema patients and controls. Our findings highlight significant differences, particularly in overall, metabolic, and mental health, as well as pain perception. Although participants were group-matched for age, gender, and BMI, the fact that in the literature [4,9,16] and in our cohort, many lipedema patients struggle with higher BMI values makes these significant differences even more remarkable. This emphasizes the distinct nature of lipedema beyond mere obesity-related issues, even in advanced stages where many patients present with additional obesity, blurring the differences and making it more difficult to distinguish between these two entities. Furthermore, it is important to acknowledge that BMI does not differentiate between the localization of enlarged adipose tissue, which is a significant concern in the context of lipedema. Therefore, a BMI greater than 30 kg/m^2^ in lipedema patients may not indicate traditional obesity following the WHO criteria, bearing completely different implications for general, metabolic, and mental health.

Compared with controls, patients with lipedema had lower incidences of hypertension, high blood lipids, and diabetes. However, they rated their overall health lower. This is in line with findings from other studies [11,25,26,27] and strengthens the hypothesis that the frequently emphasized gynoid fat distribution (hips, buttocks, legs) in lipedema could have a positive effect on metabolic health [12,27].

Patients with lipedema in our study experienced higher levels of pain, greater disability in daily activities due to pain, and higher severity and prevalence of depression. A significant positive correlation between the use of physiotherapy services and patients with lipedema was demonstrated, emphasizing the logical consequence of more physical complaints in patients with lipedema, making physiotherapy necessary [28]. The main objective of physiotherapy is to alleviate lower extremity symptoms and reduce disability and functional limitations, ultimately enhancing the quality of life of patients and preventing disease progression [29].

Pain remains a cardinal symptom of lipedema and is critical in distinguishing it from obesity. It is a complex manifestation that extends beyond mere quantification on pain scales. Pain associated with lipedema is linked to lipedema fat, and various hypotheses have been proposed, including allodynia and exaggerated sympathetic signaling [5,30,31]. In line with prior studies [31,32], patients with lipedema in our cohort most frequently described the pain with the adjectives pressing, dull, and pulling potentially revealing the underlying pathophysiology. The pressing and dull pain points to a chronic inflammatory process, likely due to the accumulation of adipose tissue exerting pressure on joints and tissues. This type of pain aligns with nociceptive characteristics, resulting from the stimulation of nociceptors by physical stress or damage. In contrast, neuropathic pain is often described as burning, shooting, stabbing, prickling, and electric shock-like pain, which some of our patients also reported. The varied nature of pain in lipedema, combining aspects of nociceptive pain, neuropathic pain, and possibly central sensitization [33], reflects the complex interplay of mechanical pressure, inflammation, and central pathologies. An important differential diagnosis in this context is lipomatosis dolorosa or Dercum’s disease—a condition primarily known for painful fat accumulation in the form of painful lumps. However, clinical presentation including pattern of affliction, localized pain, inheritance, and associated conditions, e.g., lipomas or angiolipomas, may help in distinguishing between these conditions [3]. Understanding these pain characteristics is vital for tailoring effective treatment strategies. A correlation between the stage of lipedema and the severity or intensity of pain could not be demonstrated. As previously described in the literature [30], there was a significant correlation between pain and the degree of depression measured by the PHQ-8 questionnaire. Currently, pharmacological options for the management of lipedema-associated pain are inadequate. While physical therapy provides temporary relief [34], it does not address the underlying causes of pain. In contrast, tumescent liposuction has demonstrated long-term effectiveness in pain reduction, suggesting that the removal of lipedema fat can alleviate pain symptoms [5,35,36]. The insights from patients with lipedema regarding triggers and alleviating factors for pain show the promise of a multidisciplinary treatment approach with a combination of physical therapies, lifestyle adjustments, and psychological strategies, particularly in the absence of a scientifically elucidated mechanism for the origin of pain in lipedema. The importance of the subjective experiences of affected patients shows the need to develop a specific questionnaire for patients with lipedema. The diversity in current research methodologies—including varied inclusion criteria, definitions, baseline assessments, and outcome measures—impedes comparisons and consensus. In addition to medical history (demographics, key comorbid conditions, work status, diet, and physical activity) and physical examination items (e.g., body mass index, waist–hip ratio), lipedema questionnaires should include the most relevant health-related quality-of-life domains from the perspective of both clinicians and patients. Ideally, the items should also be able to assist in the diagnostic criteria [12]. This inclusion would facilitate a faster consensus on particularly problematic points using the Delphi method [37]. Kloosterman et al. [4] were the first to apply the International Classification of Functioning, Disability and Health (ICF) of the World Health Organization (WHO) as a framework to investigate lipedema. The ICF focuses on areas such as body functions, body structures, and activities and participation. The Patient-Reported Outcomes Measurement Information System (PROMIS) framework is an initiative of the National Institutes of Health that uses the WHO definition of health, which is described as physical, mental, and social health. Each category includes different domains such as physical function, pain intensity, and depression. In our study, we applied the German Health Update (GEDA 2019/2020-EHIS) questionnaire, which is based on four categories: health status, healthcare, health determinants, and socioeconomic variables [22]. Studies measuring longitudinal outcomes after dietary interventions [38] or liposuction [36,39] have also used different outcome measurements. In the study of Di Renzo et al. [38], the quality of life was assessed in patients using the Fibromyalgia Impact Questionnaire (FIQ). This questionnaire includes items that evaluate physical functioning, work status, depression, anxiety, sleep, pain, stiffness, fatigue, and well-being. However, specific details regarding the application or adaptation of the FIQ for the lipedema study group were not provided. In another study [39], the quality of life before and after liposuction was measured using a visual analog scale (VAS). This scale, ranging from 0 to 10, allowed patients to rate the severity of various symptoms, including spontaneous pain, sensitivity to pressure, feeling of tension, bruising, cosmetic impairment, and overall impairment to their quality of life. In their retrospective chart review, Wright et al. [36] were the first to apply PROMIS to lipedema, together with bioelectrical impedance analyses, knee kinematics, gait, physical examinations, and Short Form-36 questionnaires, to analyze the outcomes of lipedema reduction surgery. Essentially, there are different questionnaires, but there is no standardized, universally accepted questionnaire for patients with lipedema.

Our study is the first to show differences in the most important health domains between lipedema patients and BMI-matched controls from the general population. Although other studies [16,17] have found similar results, their reference population was not matched by BMI. Other approaches to investigate quality of life had patients with fibromyalgia [8] or Dercum’s disease (adiposis dolorosa) [40] as control groups. Despite the important results for lipedema research and the representative lipedema group, our study has a limitation in the selection of controls. Possible allocation biases between intervention and control groups were addressed through 1:5 matching, notwithstanding the exclusion of this disease from the German Health Update.

## 5. Conclusions

In conclusion, this is the first study to show significant differences in the main domains of patients with lipedema compared with sex-, BMI-, and age-matched controls with lower prevalence of hypertension and high blood lipids in the lipedema group. Furthermore, lipedema patients reported poorer overall health, greater use of physiotherapy, higher depression rates, fewer close contacts, and more severe pain and pain-related disability compared to matched controls. Our results reaffirm the need for a validated lipedema-specific questionnaire that evaluates both momentary characteristics and longitudinal outcomes. Future research should focus on developing such a questionnaire, together with exploring multidisciplinary treatment strategies to enhance the quality of life for patients with lipedema. This progression is critical for advancing our understanding and the management of lipedema patients.

## Figures and Tables

**Table 1:** Inclusion criteria for patients with lipedema (German S1-guidelines).

Female
Bilateral and symmetrical enlargement of the limbs
Minimal pitting edema
Pain and tenderness on palpation
Mild bruising
Persistent enlargement after elevation of the extremities or weight loss
Negative Kaposi–Stemmer sign

**Table 2:** Characteristics of patients with lipedema and matched controls.

Clinical Characteristics		Lipedema *n* = 39	Matched Controls *n* = 195
Sex			
Male	*n* (%)	0 (0.0)	0 (0.0)
Female	*n* (%)	39 (100.0)	195 (100.0)
	Mean (±SD)	46.8 (±10.6)	48.2 (±8.2)
Age	18–29	1 (2.6)	5 (2.6)
Years Group	30–44	16 (41.0)	80 (41.0)
	45–64	22 (56.4)	110 (56.4)
	65+	0 (0.0)	0 (0.0)
	Mean (±SD)	35.6 (±7.6)	35.0 (±6.7)
Body mass index (BMI, kg/m^2^)	BMI 18.5–24.9	2 (5.1)	10 (5.1)
Level Group	BMI 25–29.9	4 (10.3)	20 (10.3)
	BMI 30–34.9	12 (30.8)	60 (30.8)
	BMI 35–39.9	8 (20.5)	40 (20.5)
	BMI 40 or greater	13 (33.3)	65 (33.3)
Lipedema stage	St. I	9 (23.1)	N.A.
	St. II	21 (53.8)	N.A.
	St. III	9 (23.1)	N.A.
Onset of lipedema	Puberty	21 (53.8)	N.A.
	Pregnancy	5 (12.8)	N.A.
	Menopause	2 (5.1)	N.A.
	Other	11 (28.2)	N.A.
Family history of lipedema	Positive	25 (64.1)	N.A.

Values are mean (±standard deviation) or absolute values (percentages). N.A., not applicable.

**Table 3:** Details of pain characteristics in patients with lipedema (German Pain Questionnaire).

Characteristics of Pain	Detailed Description	N = 39
Pain patterns		
	Presence of pain at rest	31 (79.5)
	Presence of pain with movement	34 (87.1)
Sensation of pain	dull	20 (51.3)
	oppressive	25 (64.1)
	throbbing	12 (30.8)
	knocking	5 (12.8)
	stabbing	17 (43.6)
	pulling	22 (56.4)
	hot	9 (23.1)
	burning	15 (38.5)
	miserable	17 (43.6)
	horrible	9 (23.1)
	awful	17 (43.6)
Intensity (range 0–10)	At the moment	6 (±2.5)
	Within the last 4 weeks	
	mean	4 (±1.0)
	maximum	8 (±2.3)
	Tolerable pain level with adequate treatment	4 (±2.0)

Values are mean (±standard deviation) or absolute values (percentages).

## Data Availability

Derived data supporting the findings of this study are available from the corresponding author upon reasonable request.
